# Research hotspots and trends in the application of Chinese herbal medicine for cancer pain management: a bibliometric study

**DOI:** 10.3389/fmed.2026.1889170

**Published:** 2026-07-21

**Authors:** Ning Lu, Jianhua Li, Shanshan Jiang, Qing Hou, Lanfang Shen, Lu Zhang, Junfan Wei

**Affiliations:** 1 Department of Pharmacy, Nanjing Central Hospital, Nanjing, China; 2 Department of Nursing, Nanjing Central Hospital, Nanjing, China; 3 Department of Management Office of Hexi Outpatient, Nanjing Central Hospital, Nanjing, China; 4 Department of Emergency, Nanjing Central Hospital, Nanjing, China

**Keywords:** bibliometric analysis, cancer pain management, Chinese herbal medicine, integrative medicine, knowledge visualization

## Abstract

**Background:**

Chinese herbal medicine has been increasingly used as a complementary approach for cancer pain management. However, the global research landscape, collaboration patterns, and emerging trends in this field have not been systematically characterized.

**Methods:**

Publications related to Chinese herbal medicine for cancer pain management were retrieved from the Web of Science Core Collection and Scopus databases. Bibliometric analyses were conducted using CiteSpace, VOSviewer, and Bibliometrix to evaluate publication trends, country and institutional collaborations, author networks, co-citation relationships, and keyword co-occurrence patterns.

**Results:**

Research output increased steadily over time, with China leading in publication volume and the United States demonstrating strong international collaboration. Highly cited publications formed the intellectual foundation of complementary pain management and evidence-based integrative oncology research. Keyword analysis identified major research themes in cancer pain management, quality of life, integrative oncology, and evidence-based medicine, while “network pharmacology,” “mechanism,” and “meta-analysis” emerged as recent research hotspots.

**Conclusion:**

This bibliometric analysis provides a comprehensive overview of the knowledge structure, research hotspots, and development trends in Chinese herbal medicine for cancer pain management. The findings highlight the intellectual foundation and emerging themes of this research field, and may provide useful insights to guide future evidence-based and collaborative research in integrative oncology.

## 1 Introduction

Pain is one of the most common and distressing symptoms experienced by patients with cancer, significantly impacting their quality of life, mental health, and the overall clinical treatment outcomes (1, 2). Despite advances in traditional pain management strategies (primarily opioid therapy), many patients still fail to achieve adequate pain control due to adverse reactions, drug tolerance, and limited efficacy (3). Consequently, there is growing interest in complementary and integrative approaches to enhance the effectiveness and safety of cancer pain management (4).

In recent years, Chinese herbal medicine has been widely used as an adjunctive therapy for cancer due to its holistic and multi-targeted approach (5). Chinese herbal medicines are often combined with conventional drug therapies to alleviate symptoms, reduce treatment-related side effects, and improve patients' quality of life (6). Increasing clinical and experimental evidence suggests that Chinese herbal medicine may alleviate cancer pain, improve quality of life, and enhance the efficacy of conventional analgesic therapies (7, 8). Growing interest in the mechanisms and clinical applications of Chinese herbal medicine has led to a rapid increase in publications in this field (9). At the same time, research on Chinese herbal medicine in the field of oncology has garnered growing attention, with a mounting body of clinical evidence supporting its role in managing cancer symptoms and improving quality of life (10).

Although publications on Chinese herbal medicine for cancer pain have increased substantially in recent years, the overall research landscape remains fragmented and has not been systematically characterized (11, 12). Differences in research focus, study design, and thematic orientation make it difficult to gain a clear and comprehensive understanding of developments in this field (13). Previous studies have mainly focused on clinical efficacy, quality of life, or palliative care outcomes, whereas little attention has been paid to the overall knowledge structure, collaboration networks, research hotspots, and developmental trends of this field (11, 14, 15). Consequently, a comprehensive understanding of how this field has evolved, who the major contributors are, and which topics are driving current and future research is still lacking.

Bibliometric analysis is a quantitative approach for systematically evaluating the development of a scientific field by mapping publication patterns, collaboration networks, citation relationships, and research hotspots. Unlike conventional reviews that mainly summarize clinical evidence, bibliometric analysis provides a macroscopic perspective on the evolution of scientific knowledge and helps identify influential contributors, emerging themes, and potential research directions. Therefore, bibliometric analysis is particularly well suited to addressing the current lack of a comprehensive overview of research on Chinese herbal medicine for cancer pain management.

Accordingly, this study conducted a comprehensive bibliometric analysis of publications on Chinese herbal medicine for cancer pain management retrieved from the Web of Science Core Collection (WOSCC) and Scopus databases. Using CiteSpace, VOSviewer, and Bibliometrix, we aimed to characterize publication trends, identify major contributing countries, institutions, authors, and journals, map collaboration and co-citation networks, and reveal keyword co-occurrence patterns and emerging research hotspots. By providing a systematic overview of the global research landscape, this study may help researchers identify future research priorities, facilitate academic collaboration, and support the evidence-based development of Chinese herbal medicine in cancer pain management.

## 2 Materials and methods

### 2.1 Data sources and search strategy

**Figure 1:**
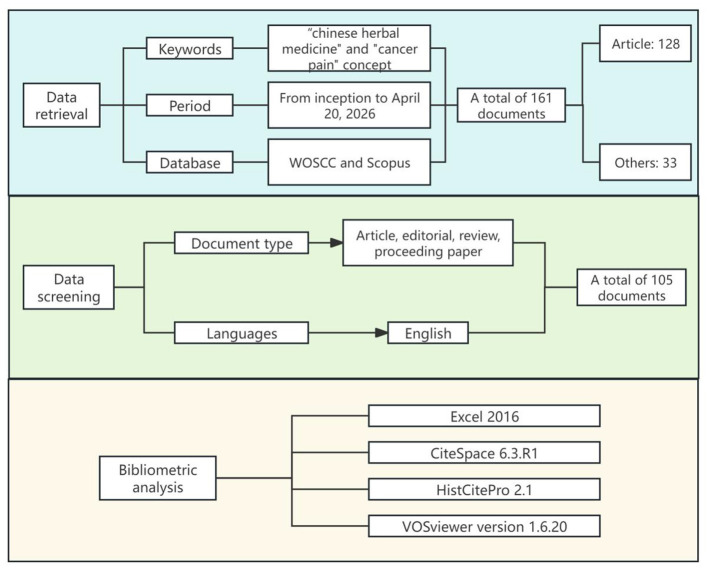
Flow chart of literature screening.

The Web of Science Core Collection (WOSCC), provided by Clarivate, is one of the most authoritative and widely used databases for scientific literature and citation analysis. Scopus, developed by Elsevier, is also one of the largest multidisciplinary databases of peer-reviewed abstracts and citations, covering natural sciences, medicine, social sciences, and the humanities. In this study, WOSCC and Scopus were selected because they provide broad journal coverage, standardized bibliographic records, and relatively complete citation metadata, making them suitable for bibliometric mapping, cross-database integration, and visualization analysis. Other databases, such as PubMed, Embase, CNKI, and regional databases, were not included because of differences in indexing scope, citation metadata availability, language coverage, and data export formats, which may increase heterogeneity during data merging and affect the comparability of bibliometric indicators. Accordingly, this study chose both databases as data sources and ultimately established the literature search strategy in the WOSCC database as: TS = (“chinese medicinal herb^*^” OR “chinese herb^*^” OR “chinese herbal medicine^*^” OR “chinese herbal” OR “herb^*^”) AND TS = (“cancer pain^*^” OR “cancer-induced pain” OR “tumor pain” OR “cancerous pain” OR “cancer ache” OR “cancer pain” OR “cancer-associated pain^*^” OR “cancer associated pain^*^” OR “cancer-related pain^*^” OR “cancer related pain^*^” OR “neoplasm-associated pain^*^” OR “neoplasm associated pain^*^” OR “neoplasm-related pain^*^” OR “neoplasm related pain^*^” OR “oncological pain^*^” OR “oncology pain^*^” OR “tumor-associated pain^*^” OR “tumor associated pain^*^” OR “tumor-related pain^*^” OR “tumor related pain^*^”). The literature search query in the Scopus database is: TITLE-ABS (“chinese medicinal herb^*^” OR “chinese herb^*^” OR “chinese herbal medicine^*^” OR “chinese herbal” OR “herb^*^”) AND TITLE-ABS (“cancer pain^*^” OR “cancer-induced pain” OR “tumor pain” OR “cancerous pain” OR “cancer ache” OR “cancer pain” OR “cancer-associated pain^*^” OR “cancer associated pain^*^” OR “cancer-related pain^*^” OR “cancer related pain^*^” OR “neoplasm-associated pain^*^” OR “neoplasm associated pain^*^” OR “neoplasm-related pain^*^” OR “neoplasm related pain^*^” OR “oncological pain^*^” OR “oncology pain^*^” OR “tumor-associated pain^*^” OR “tumor associated pain^*^” OR “tumor-related pain^*^” OR “tumor related pain^*^”). The search period spanned from the database inception to April 20, 2026, yielding a total of 161 articles. To ensure the accuracy and reliability of the data, two researchers independently screened the retrieved literature according to predefined inclusion and exclusion criteria. The inclusion criteria were as follows: (1) the research focused on traditional Chinese herbal medicine in the management of cancer pain; and (2) the publication language was English. The exclusion criteria were: (1) conference abstracts, letters, theses, and other non-article literature types; (2) duplicate publications; and (3) errata. After the independent screening process, the results were cross-checked, and any disagreements were resolved through discussion with a third researcher. Ultimately, 105 English-language documents were included. The literature screening flowchart is shown in Figure 1.

### 2.2 Data standardization

The qualifying publications were exported as “Full Record and Cited References” in Plain Text File format and saved as “download^*^.txt.” The resulting plain-text files were imported into CiteSpace, Bibliometrix, and VOSviewer for visualization analysis.

The retrieved records were first screened according to the predefined search criteria in the WOSCC and Scopus databases. To eliminate duplicates between Web of Science and Scopus, the two datasets were merged, and CiteSpace's built-in deduplication function was applied. This function detects overlapping records using multiple metadata fields, including title, author, journal, and publication year. Records with identical or highly similar metadata were marked as potential duplicates.

Following automated deduplication, two researchers independently reviewed the flagged records to verify duplicates and resolve any inconsistencies. For each duplicated article, only one record was retained in the final dataset for subsequent analysis.

To improve consistency and accuracy, synonymous terms were standardized by constructing a CiteSpace alias file and a VOSviewer thesaurus file. In addition, irrelevant or meaningless terms, such as “123” and “a” were removed using a customized exclusion file.

### 2.3 Data analysis and visualization

This study utilized bibliometric analysis to investigate publications concerning the use of traditional Chinese herbal medicine in managing cancer-related pain. The tools applied for this analysis included CiteSpace 6.3.R1, Bibliometrix, VOSviewer, and Excel 2021, each fulfilling specific analytical functions.

CiteSpace, developed by American scholar Chaomei Chen (16), is a Java-based software designed for scientific bibliometric and visualization analysis. It is commonly applied to reveal knowledge structures, track developmental trends, and identify emerging research hotspots in academic literature. In this study, CiteSpace was utilized to consolidate literature data from multiple databases, enhancing information quality through integration and deduplication.

Bibliometrix is an open-source bibliometric analysis tool developed by Aria and Cuccurullo et al. (17), built on the R software platform. It supports citation analysis, co-occurrence analysis, the construction of collaboration networks, and the identification of topic evolution. Its graphical interface module, Biblioshiny, allows for interactive operations and visualization of results, making the tool widely utilized in constructing scientific knowledge graphs and evaluating research performance.

VOSviewer, developed by Nees Jan van Eck and Ludo Waltman at Leiden University in 2010 (18), is widely used for bibliometric mapping and visual analysis. Although the software was initially created to process bibliographic data from scientific publications, it is also suitable for exploring other forms of relational network data. It can generate co-occurrence, co-authorship, co-citation, and coupling networks, helping researchers examine connections among different elements in a research field. In the present study, VOSviewer was applied to visualize collaboration and knowledge networks involving countries/regions, institutions, authors, journals, references, and keywords. It was also used to assess the centrality of countries/regions and calculate total link strength (TLS). In the visual maps, each node represents an analyzed item, and the links between nodes indicate relationships or collaborations. Thicker links denote stronger connections and higher TLS values. Through these visualizations, the overall research profile, major contributors, collaborative relationships, research hotspots, and potential future trends in the application of traditional Chinese herbal medicine for cancer pain management could be presented more clearly and intuitively.

The H-index was used as an indicator to evaluate the scholarly impact of authors, institutions, and journals (19). Journal Impact Factor (IF), released annually by Clarivate, is another widely recognized index for assessing journal influence. It reflects the average citation frequency of articles published in a journal during the previous 2 years in the current reporting year (20). Journal Citation Reports (JCR), also provided by Clarivate Analytics, offers comprehensive citation-based information for evaluating the academic performance and influence of journals worldwide (21). In this study, the 2024 IF, JCR quartile, and H-index data were obtained from the Web of Science database. In addition, Microsoft Excel 2021 was applied to generate bar charts showing publication trends and to conduct exponential curve fitting.

## 3 Results

### 3.1 Overview of main information and annual publications trend

**Figure 2:**
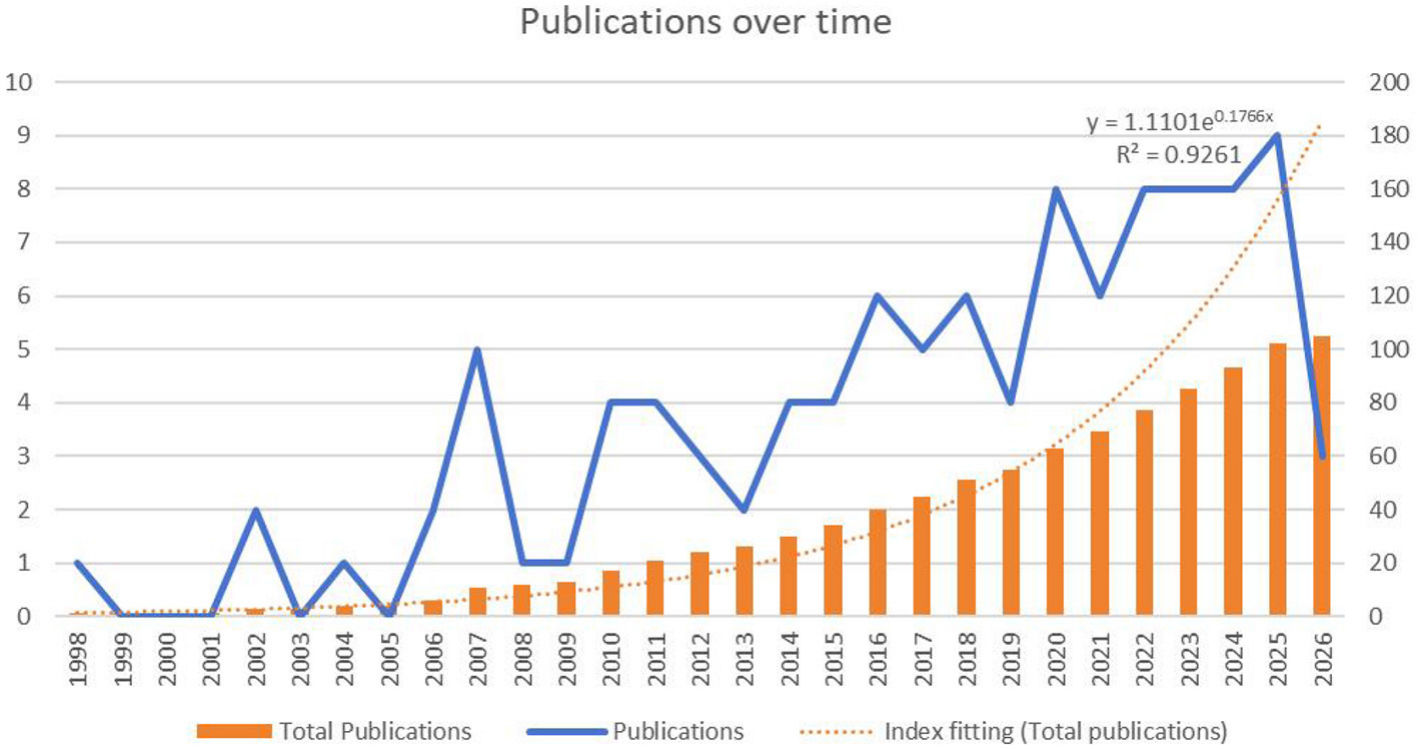
Annual publication trends in research on Chinese herbal medicine for cancer pain management. The blue line represents the annual number of publications, whereas the orange bars indicate the cumulative publication output. The dashed curve represents the exponential fitting of cumulative publications, illustrating the overall growth trend of research activity during the study period.

To assess the temporal development and research activity in this field, annual publication trends were analyzed. The literature review included a total of 105 articles, of which 74 (70.48%) were original research studies and 27 (25.71%) were reviews. For details, please refer to Supplementary material. Academic interest in the application of traditional Chinese herbal medicine for cancer pain management first emerged in 1998 with only one published paper. Overall, the publication trend can be divided into three stages. The first stage (1998–2005) was the exploratory period, during which annual publications were very limited and appeared intermittently. The second stage (2006–2013) represented a phase of steady development, with the number of publications gradually increasing and indicating growing scholarly attention to this topic. The third stage (2014–2026) was characterized by rapid expansion, during which annual publication output increased markedly and reached its highest level in 2025. Meanwhile, the cumulative number of publications showed a continuous upward trend over the entire study period. The exponential fitting curve demonstrated a high goodness of fit (y = 1.1101e^0.1766x^, *R*^2^ = 0.9261), indicating an accelerating growth trajectory in this research field. The decrease observed in 2026 should be interpreted with caution because the data for that year may not yet be complete. Overall, these results indicate that research on Chinese herbal medicine for cancer pain management has shifted from an emerging topic to a rapidly expanding field of academic interest. For details, please refer to Figure 2.

### 3.2 Distribution of countries/regions and institutions

To explore the global distribution and collaboration patterns of research in this field, the contributions of countries/regions and institutions were analyzed. A total of 22 countries/regions contributed to the research in this field. Research output was highly concentrated in a small number of countries, with China and the United States emerging as the leading contributors and together accounting for nearly 70% of the total publications. Other countries contributed substantially fewer publications, indicating a geographically uneven distribution of research activity. These findings indicate that the global research landscape is led mainly by China and the United States, and broader international collaboration remains uneven. A detailed breakdown of publication numbers, percentages, TLS, and first appearance time is shown in Table 1.

**Table 1:** The countries/regions of publication volume.

Rank	Countries/regions	Publications	Percentage	TLS	First appearance time
1	China	44	41.90	17	2007
2	United States	29	27.62	22	2002
3	South Korea	7	6.67	2	2015
4	Australia	6	5.71	9	2018
5	India	6	5.71	3	1998
6	Italy	5	4.76	4	2014
7	Canada	4	3.81	4	2015
8	Germany	4	3.81	4	2016
9	Iran	4	3.81	1	2018
10	Israel	4	3.81	2	2013
11	England	3	2.86	5	2018
12	Japan	3	2.86	1	2016
13	Taiwan	3	2.86	2	2011
14	Hong Kong	2	1.90	4	2020
15	Spain	2	1.90	4	2011
16	Burkina Faso	1	0.95	2	2020
17	Norway	1	0.95	2	2023
18	Poland	1	0.95	1	2025
19	Saudi Arabia	1	0.95	3	2022
20	Singapore	1	0.95	1	2012
21	Ukraine	1	0.95	3	2022
22	Vietnam	1	0.95	2	2026

**Figure 3:**
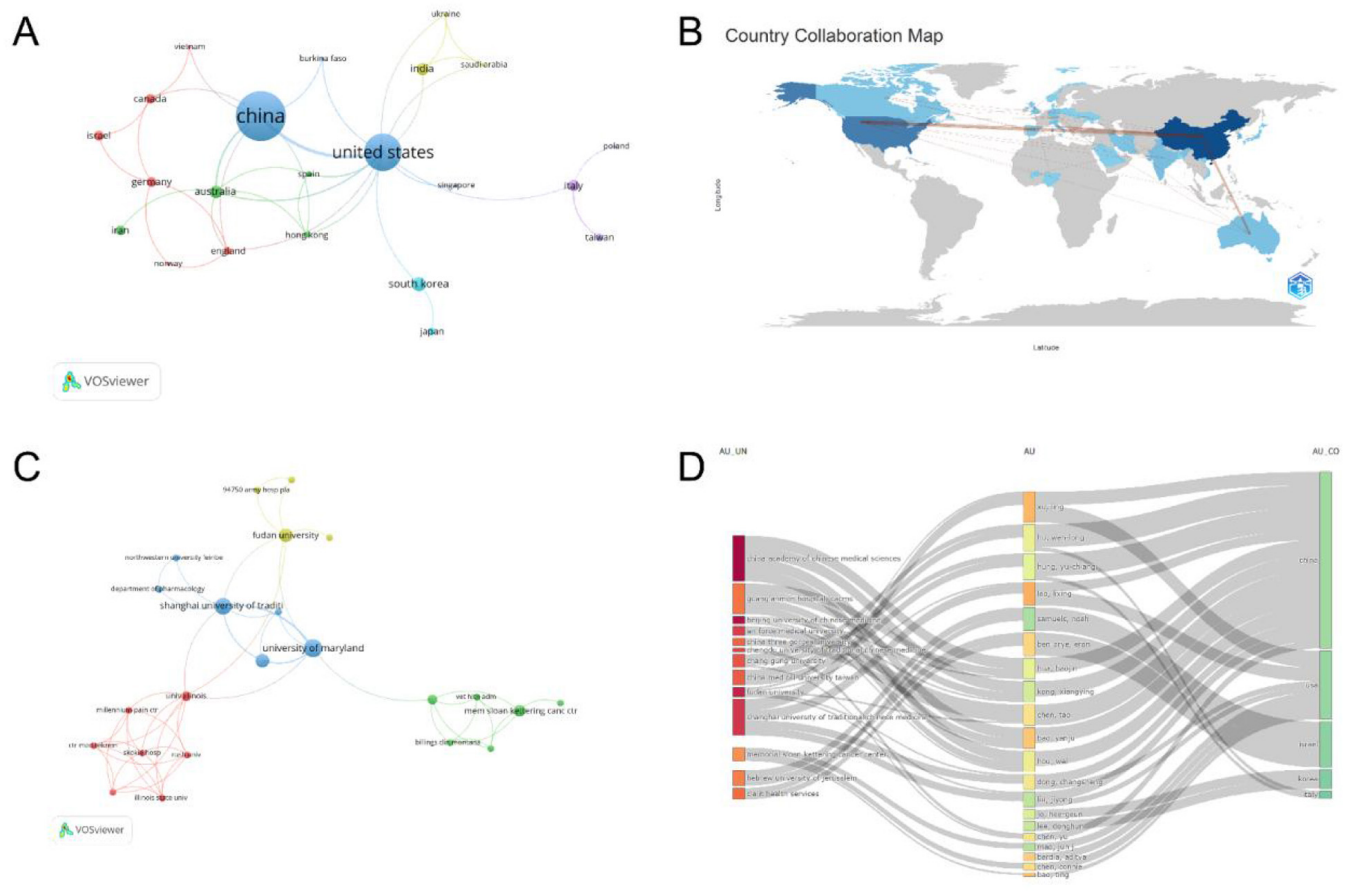
Global collaboration among countries/regions and institutions. **(A)** Network visualization of collaboration among countries/regions. Each node represents a country or region, with node size proportional to the number of publications. Links between nodes indicate collaborative relationships, and thicker links represent stronger collaboration. **(B)** Country collaboration map. Different colors indicate varying levels of international collaborative activity. **(C)** Network visualization of collaboration among institutions. **(D)** Three-field plot visualization the relationships between institutions, authors, and countries.

Figure 3A illustrates the collaborative network among countries/regions based on VOSviewer. In this network, node size represents the number of publications, while the thickness of the connecting lines indicates the strength of collaboration between countries/regions. China and the United States were the most influential and central nodes in the collaboration network, showing extensive cooperation with multiple countries/regions. Strong collaborative relationships were observed between the United States and countries such as India, South Korea, Italy, and Australia, while China maintained active collaborations with Canada, Australia, and several Asian and European countries/regions. These findings suggest that international cooperation in this field has become increasingly interconnected, with China and the United States playing dominant roles in promoting global research collaboration. Figure 3B presents the global country collaboration map, in which different colors indicate varying levels of collaborative activity among countries/regions. The map demonstrates that collaborations were mainly concentrated in North America, Asia, and Europe. China and the United States formed the primary hubs of international cooperation and established extensive research connections with countries including Australia, India, Italy, South Korea, Canada, and several European countries. Overall, the results indicate that cross-national collaboration has become an important driving force for the development of research in this field.

In terms of institutional collaboration, a total of 205 institutions participated in research on the Application of Traditional Chinese Herbal Medicine for Cancer Pain Management. Table 2 lists the top 10 institutions by number of published papers. The most productive institutions were concentrated in China and the United States, indicating that research activity is concentrated within a limited number of academic centers (Table 2). The institutional collaboration network (Figure 3C) illustrates the collaborative relationships among the major research institutions in this field. Node size represents the number of publications produced by each institution, while the connecting lines indicate the strength of collaboration between institutions. Fudan University, Shanghai University of Traditional Chinese Medicine, and the University of Maryland emerged as major nodes in the network, indicating their important contributions and central roles in institutional cooperation. In addition, active collaborations were observed among institutions from China and the United States, including Memorial Sloan Kettering Cancer Center, Beijing University of Chinese Medicine, and Northwestern University Feinberg School of Medicine. These findings indicate that leading institutions have established active collaborative networks, although cooperation remains concentrated among a limited number of institutions.

**Table 2:** The top 11 institution of publication volume.

Rank	Institution	Country	TLS	Publications
1	University of Maryland	United States	12	7
2	Shanghai University of Traditional Chinese Medicine	China	12	6
3	Beijing University of Chinese Medicine	China	13	5
4	Fudan University	China	6	4
5	Second Military Medical University	China	6	4
6	Chang Gung University	Taiwan, China	12	3
7	Chengdu University of Traditional Chinese Medicine	China	4	3
8	China Academy of Chinese Medical Sciences	China	3	3
9	China Medical University	China	11	3
10	Guangzhou University of Chinese Medicine	China	5	3
11	Memorial Sloan Kettering Cancer Center	United States	5	3

Figure 3D presents a three-field plot illustrating the relationships among institutions, authors, and countries. The plot demonstrates how authors from different institutions are connected with specific countries/regions, thereby highlighting the global distribution of academic collaborations. Major institutions, including China Academy of Chinese Medical Sciences, Shanghai University of Traditional Chinese Medicine, and Memorial Sloan Kettering Cancer Center, were closely associated with productive authors and contributed substantially to international research collaborations. Most authors were affiliated with institutions from China and the United States, indicating that these two countries played leading roles in promoting scientific output and cross-national cooperation in this field.

### 3.3 Authors and co-cited authors analysis

**Figure 4:**
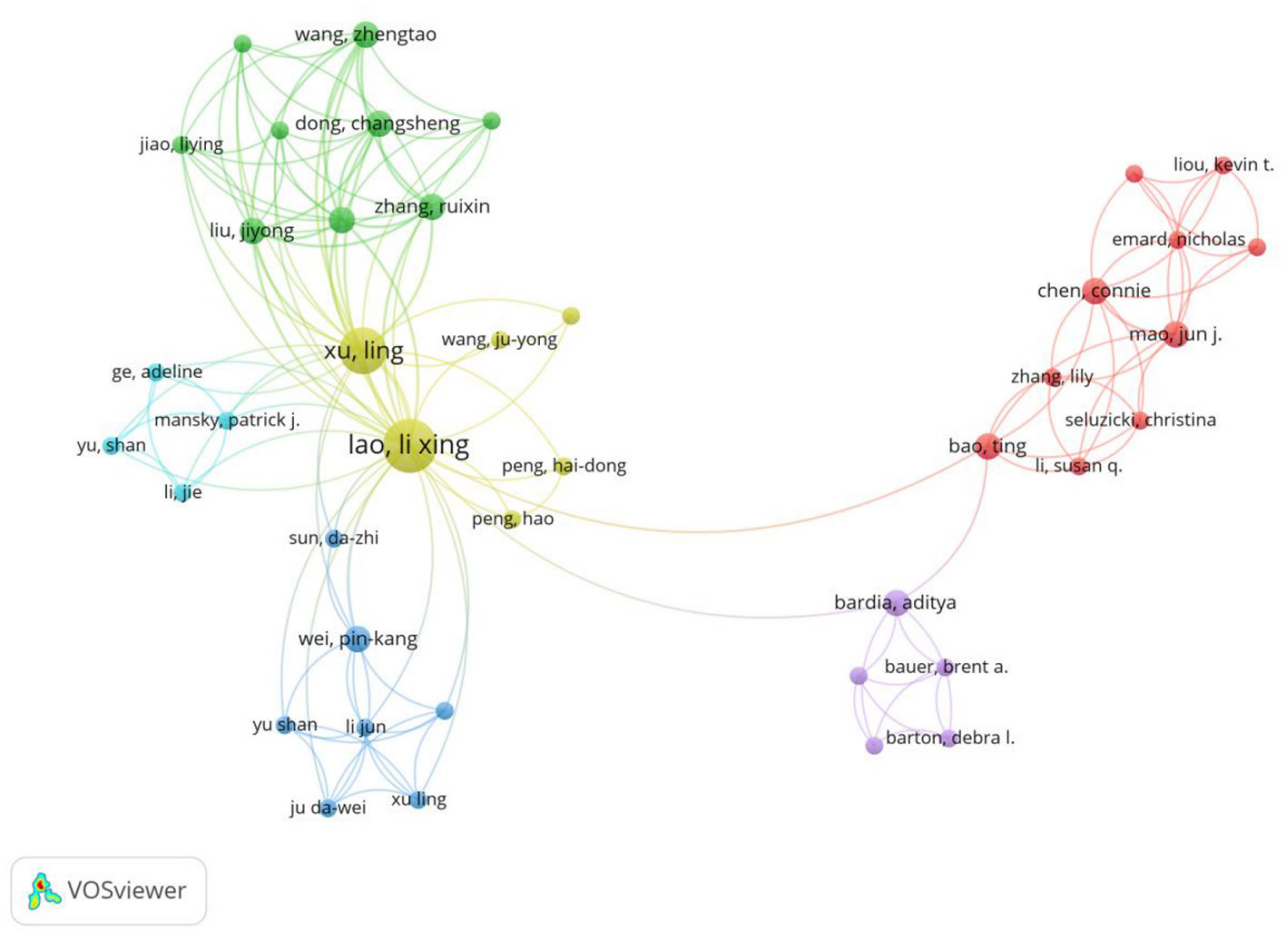
Network visualization of collaboration among authors. Each node represents an author, with node size corresponding to the number of publications. Links between nodes indicate co-authorship relationships, and different colors represent distinct collaboration clusters identified by VOSviewer. The network highlights the major collaborative groups and the central authors contributing to research on Chinese herbal medicine for cancer pain management.

To identify key contributors and collaboration patterns, author productivity and co-authorship networks were analyzed. A total of 508 authors contributed to research in this field. Lao Li Xing and Xu Ling were the most productive authors and also demonstrated strong academic influence, as reflected by their publication output, citation counts, and collaborative relationships (Table 3). Although most authors published relatively few papers, their academic influence varied considerably. Several authors with limited publication output, such as Russo Ethan B., Bardia Aditya, and Winfried Haeuser, achieved substantial citation impact, whereas others received relatively few citations. These findings suggest that publication quantity does not necessarily correspond to academic influence. The author collaboration network is shown in Figure 4, which reveals a multi-cluster cooperative structure. Overall, the network is dominated by several color-coded clusters, among which the yellow and green clusters on the left form the main collaborative core. Lao, Li Xing and Xu, Ling occupy central positions with relatively large node sizes and numerous links, indicating that they are the most active and influential collaborators in the network. The green cluster includes authors such as Wang, Zhengtao, Dong, Changsheng, Liu, Jiyong, and Zhang, Ruixin, showing dense internal connections and suggesting a stable collaborative group. The blue cluster, represented by Wei, Pin-Kang, Li Jun, Yu Shan, Ju Da-Wei, and Xu Ling, is closely connected with the central yellow cluster, indicating frequent cooperation with the core authors. In addition, the cyan cluster, including Ge, Adeline, Mansky, Patrick J., Yu, Shan, and Li, Jie, forms a smaller but relatively independent collaboration group. On the right side of the network, the red cluster is centered around Chen, Connie, Mao, Jun J., and Bao, Ting, with collaborators such as Emard, Nicholas, Liu, Kevin T., Zhang, Lily, Li, Susan Q., and Seluzicki, Christina. The dense connections within this cluster indicate strong internal cooperation, although it is relatively separated from the left-side main network. The purple cluster, represented by Bardia, Aditya, Bauer, Brent A., and Barton, Debra L., forms a small collaboration group and is mainly linked to the broader network through Bao, Ting. Notably, Lao, Li Xing and Xu, Ling serve as key bridge authors in the network, connecting multiple collaborative groups. Overall, the collaboration network is characterized by several distinct research clusters connected through a small number of core authors, indicating that author collaboration remains relatively fragmented despite the presence of influential collaborative groups.

**Table 3:** The top 32 authors of publication volume.

Rank	Author	Publications	TLS	Citations	H-index
1	Lao, Li Xing	8	38	150	47
2	Xu, Ling	6	30	144	29
3	Bao, Ting	2	7	5	26
4	Bao, Yanju	2	15	77	17
5	Bardia, Aditya	2	6	159	77
6	Ben-Arye, Eran	2	5	21	29
7	Chen, Connie	2	10	10	7
8	Chen, Tao	2	19	35	32
9	Chen, Yu	2	13	21	7
10	Chi, Yongliang	2	18	12	9
11	Delfino, Domenico V.	2	8	5	25
12	Dong, Changsheng	2	16	41	7
13	Haeuser, Winfried	2	9	89	75
14	Hou, Wei	2	15	77	22
15	Hu, Wen-Long	2	8	5	10
16	Hua, Baojin	2	15	77	28
17	Hung, Yu-Chiang	2	8	5	9
18	Jo, Hee-Geun	2	4	16	12
19	Kong, Xiangying	2	15	77	27
20	Lee, Donghun	2	4	16	17
21	Liu, Jiyong	2	16	41	42
22	Mao, Jun J.	2	10	10	53
23	Russo, Ethan B.	2	1	338	25
24	Samuels, Noah	2	5	21	19
25	Tsai, Ming-Yen	2	8	5	13
26	Wang, Juyong	2	16	41	11
27	Wang, Zhengtao	2	16	41	63
28	Wei, Pin-Kang	2	9	9	13
29	Yang, Liping	2	15	77	23
30	Yen, Hung-Rong	2	8	5	35
31	Zhang, Ruixin	2	16	41	25
32	Zhang, Wen	2	15	26	6

**Figure 5:**
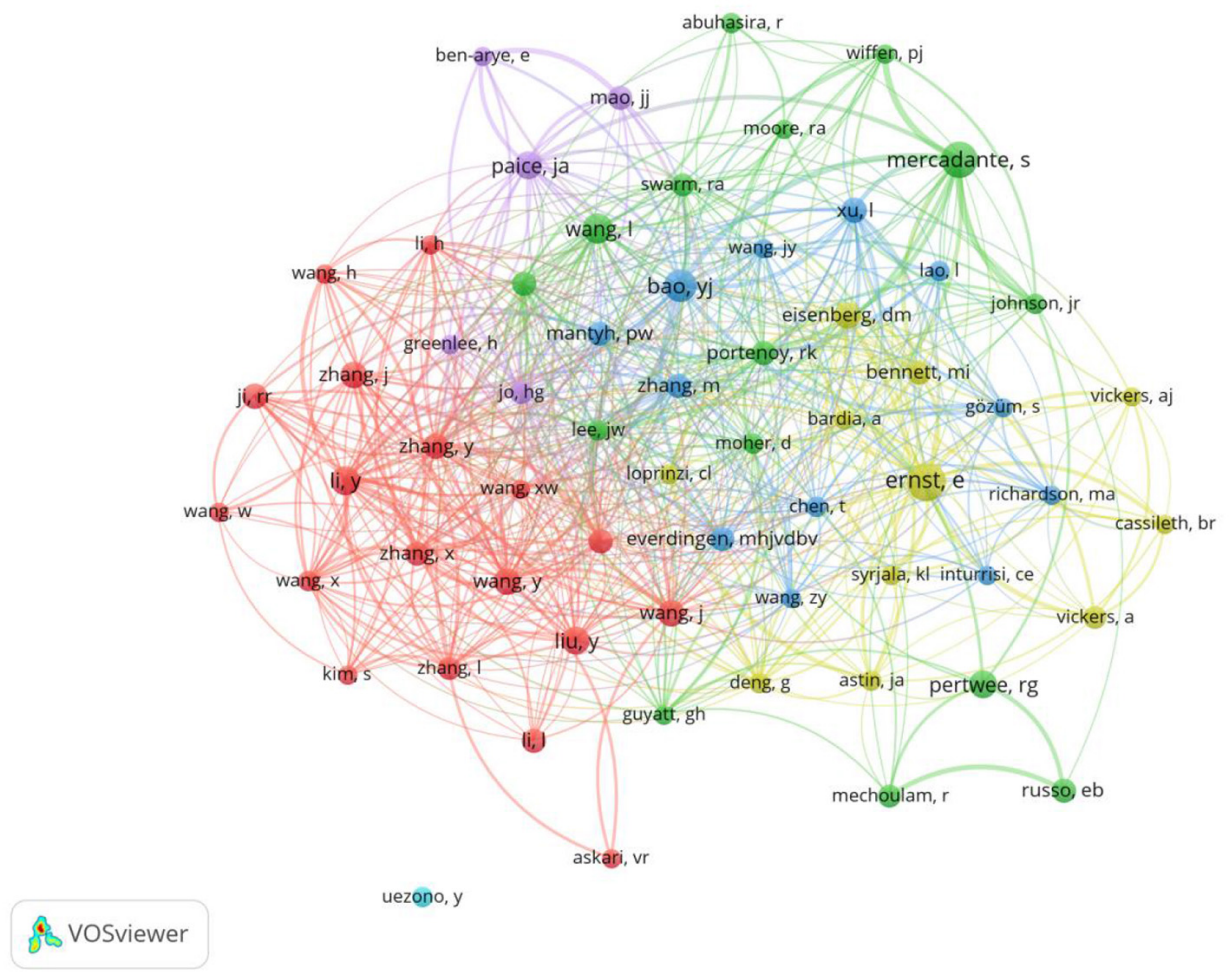
Network visualization of co-cited authors. Each node represents a co-cited author, and node size reflects co-citation frequency. Links indicate co-citation relationships, with denser connections representing stronger intellectual associations. Different colors represent clusters of authors with closely related research interests, illustrating the knowledge structure of this research field.

The top 12 co-cited authors are listed in Table 4. The results show that Ernst, E ranks first with 20 citations and a TLS of 115, indicating his important academic influence in this field. Mercadante, S follows with 18 citations and a higher TLS of 144, suggesting strong co-citation relationships with other scholars. Bao, YJ also shows a prominent position, with 15 citations and the highest TLS value 146 among the listed authors. The co-cited author network is shown in Figure 5, which visually presents the intellectual connections among key authors in this field. The network consists of several clusters, indicating that authors with close co-citation relationships tend to be grouped together. Overall, the network is highly interconnected, suggesting a relatively mature and diverse knowledge structure. Among these authors, Bao, YJ, Ernst, E, and Mercadante, S occupy prominent positions with larger node sizes and dense links, indicating their important academic influence. The red cluster on the left mainly includes authors such as Zhang, J, Li, Y, and Liu, Y, while the green and yellow clusters contain influential authors such as Mercadante, S, Pertwee, RG, and Vickers, AJ. In addition, Bao, YJ is located near the center of the network, suggesting that this author plays an important bridging role among different research groups. Overall, Figure 5 shows that the co-citation structure is characterized by multiple research clusters and several central influential authors.

**Table 4:** The top 12 co-cited authors of publication volume.

Rank	Author	TLS	Citations
1	Ernst, E	115	20
2	Mercadante, S	144	18
3	Bao, Yj	146	15
4	Wang, L	71	13
5	Li, Y	124	12
6	Liu, Y	61	11
7	Paice, Ja	86	11
8	Pertwee, Rg	29	11
9	Eisenberg, Dm	72	10
10	Wang, J	80	10
11	Wang, Y	100	10
12	Zhang, J	60	10

### 3.4 Journal and cited journal analysis

To determine the main publication sources and knowledge dissemination channels, journal and cited journal analyses were conducted. The impact factor (IF) and Journal Citation Reports (JCR) quartile rankings are commonly used to evaluate the academic influence and quality of journals. According to the JCR classification, journals ranked within the top 25% of impact factors are categorized as Q1, whereas those positioned between the top 25% and 50% are classified as Q2. Analyzing journal distribution helps identify the core journals that make substantial contributions to a specific research field (22). A total of 83 journals contributed on this filed. Research in this field was mainly published in journals specializing in pain management, pharmacology, and integrative medicine. Most of the leading journals were classified as JCR Q1 or Q2, indicating that this topic has attracted attention from well-recognized and interdisciplinary journals (Table 5). These journals represent the major publication and knowledge dissemination platforms for research on Chinese herbal medicine and cancer pain management. Overall, the results show that this research topic has received attention from both high-impact journals and interdisciplinary medical journals.

**Table 5:** The top 11 most prolific journals of publication volume.

Rank	Journal	Publications	IF (2024)	JCR (2024)
1	Journal of Pain and Symptom Management	5	3.5	Q1
2	Frontiers in Pharmacology	4	4.8	Q1
3	Integrative Cancer Therapies	4	2.8	Q2
4	Evidence-Based Complementary and Alternative Medicine	3	2.65(2021)	Q3(2021)
5	International Journal of Molecular Sciences	3	4.9	Q1
6	American Journal of Chinese Medicine	2	5.5	Q1
7	BMJ Open	2	2.3	Q2
8	Journal of Chinese Integrative Medicine	2	2.5	Q2
9	Journal of Ethnopharmacology	2	5.4	Q1
10	Journal of Pain Research	2	2.5	Q2
11	Medicine	2	1.4	Q2

**Figure 6:**
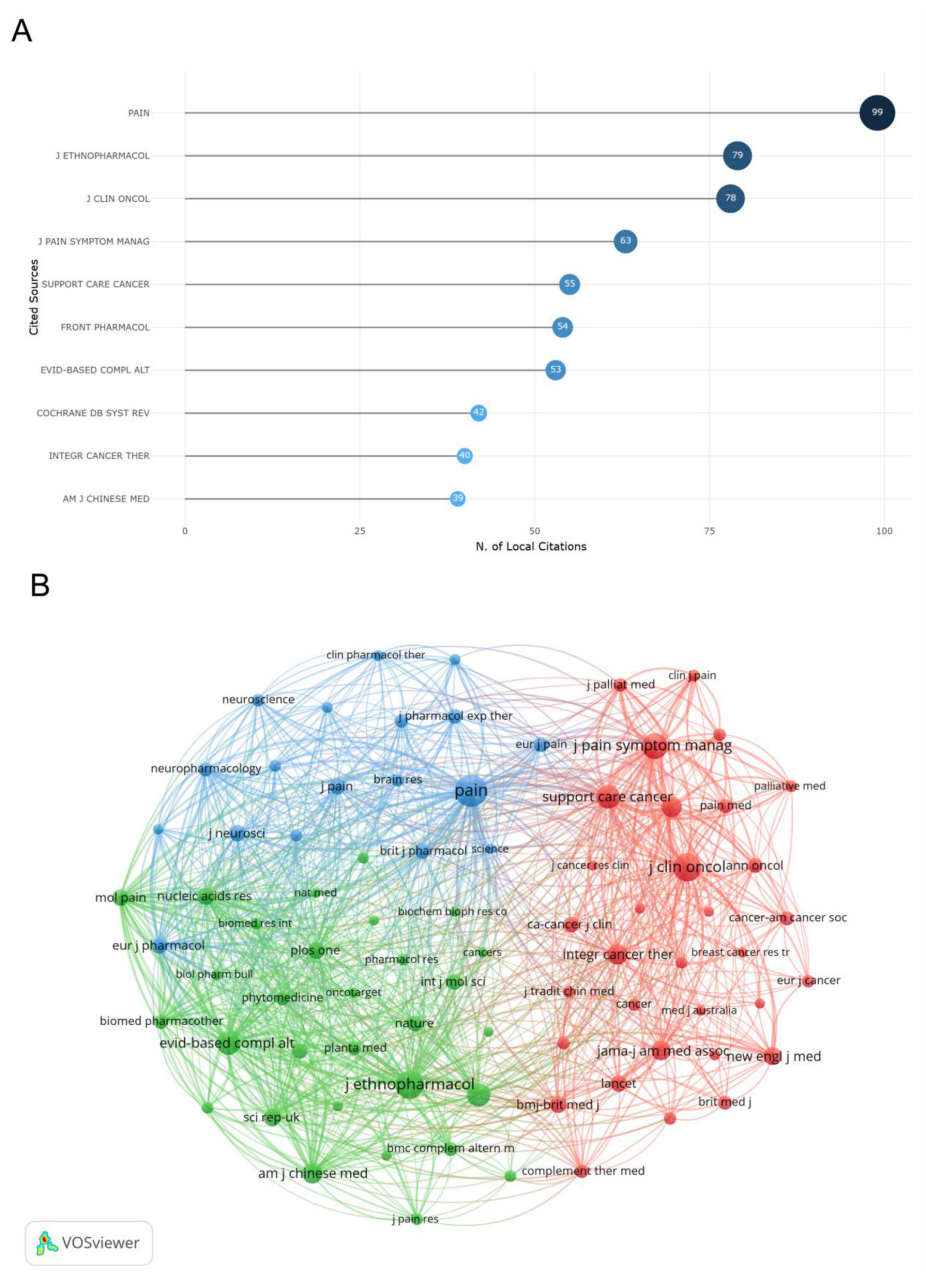
Distribution of co-cited journals. **(A)** Bar chart showing the top 10 most frequently co-cited journals ranked by local citation frequency. **(B)** Network visualization of co-cited journals. Each node represents a journal, with node size proportional to its co-citation frequency. Links indicate co-citation relationships, and different colors represent journal clusters with closely related citation patterns.

In this study, a total of 3,393 co-cited journals were identified. The most frequently co-cited journals were primarily high-impact journals in pain management, oncology, and pharmacology, reflecting the interdisciplinary knowledge base of this field (Table 6). Bar chart of top 10 most cocited journals and network visualization of co-cited journals were presented in Figure 6.

**Table 6:** The top 10 most co-cited journals.

Rank	Journal	Citations	IF (2024)	JCR (2024)
1	Pain	99	5.5	Q1
2	Journal of Ethnopharmacology	79	5.4	Q1
3	Journal of Clinical Oncology	78	41.9	Q1
4	Journal of Pain and Symptom Management	63	3.5	Q1
5	Supportive Care in Cancer	55	3.0	Q1
6	Frontiers in Pharmacology	54	4.8	Q1
7	Evidence-Based Complementary and Alternative Medicine	53	2.65 (2021)	Q3 (2021)
8	Cochrane Database of Systematic Reviews	42	9.4	Q1
9	Integrative Cancer Therapies	40	2.8	Q1
10	The American Journal of Chinese Medicine	39	5.5	Q1

### 3.5 Reference citation analysis

**Figure 7:**
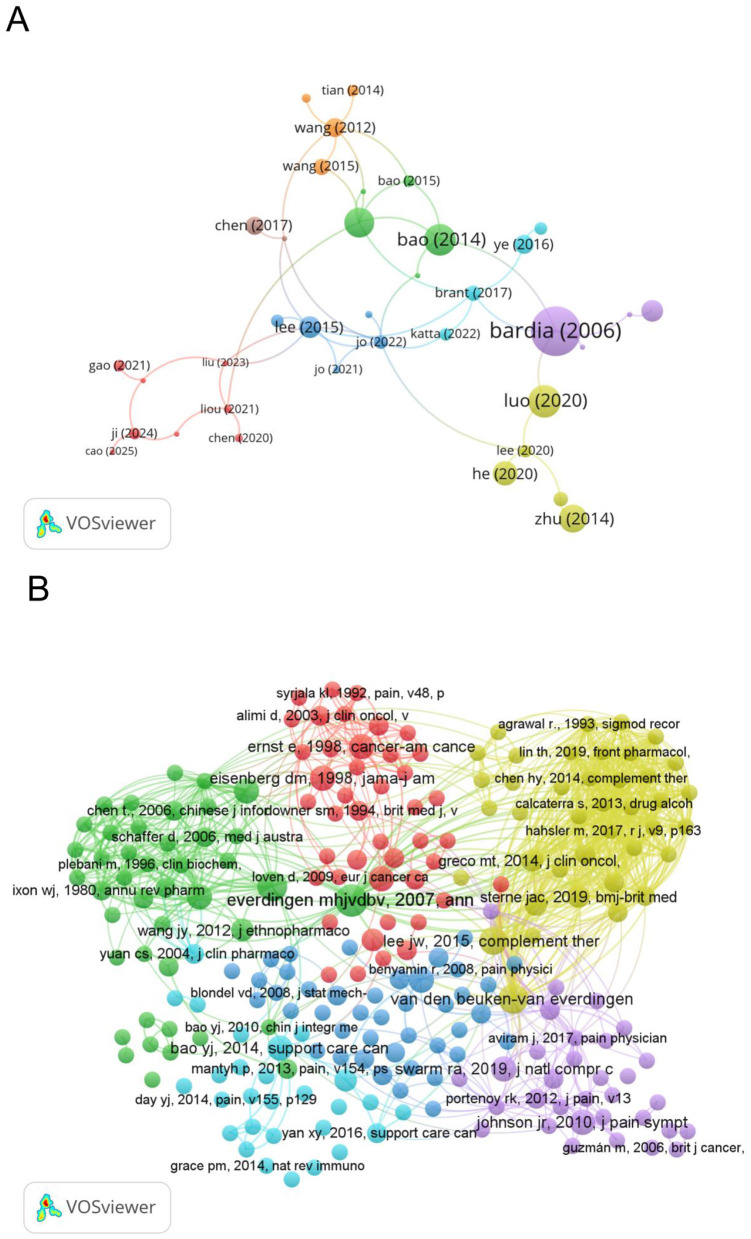
Citation and co-citation analysis of references. **(A)** Network visualization of reference citation. Each node represents a cited publication, with node size reflecting citation frequency. Links indicate citation relationships among publications. **(B)** Network visualization of co-cited references. Each node represents a co-cited reference, and links indicate co-citation relationships. Different colors represent clusters of references that constitute major knowledge domains within the field.

To identify the intellectual base and influential studies in this field, citation and citation analyses of references were performed. By examining the most frequently cited references in this field, the core themes and research priorities can be identified, which helps newcomers quickly understand the development path and future direction of the research area (23). The citation frequency of the 105 publications included in this study was analyzed using VOSviewer. The top 10 most frequently cited publications are presented in Table 7, and the citation network is shown in Figure 7A. The most cited publication, “Cannabinoids in the management of difficult to treat pain” (24), highlights cannabinoids as adjunctive options for refractory pain, expanding the scope of non-opioid analgesic strategies in pain management research. The second most cited article, “Efficacy of Complementary and Alternative Medicine Therapies in Relieving Cancer Pain: A Systematic Review” (25), assessed multiple complementary interventions and reported limited but promising evidence, while emphasizing methodological limitations in existing studies. Overall, these studies form part of the knowledge base supporting complementary pain management approaches and are closely associated with the emergence of keyword clusters related to integrative and evidence-based cancer pain research.

**Table 7:** The top 10 most frequently cited documents.

Rank	Title	Journal	Year	Citations
1	Cannabinoids in the management of difficult to treat pain	Therapeutics And Clinical Risk Management	2008	247
2	Efficacy of Complementary and Alternative Medicine Therapies in Relieving Cancer Pain: A Systematic Review	Journal of Clinical Oncology	2006	159
3	Prevalence and Treatment of Menopausal Symptoms Among Breast Cancer Survivors	Journal of Pain And Symptom Management	2002	152
4	The Cannabis sativa vs. Cannabis indica Debate: An Interview with Ethan Russo,MD	Cannabis And Cannabinoid Research	2016	91
5	Pharmacology of opioids in the treatment of chronic pain syndromes	Pain Physician	2011	89
6	Effects of Herbal Medicines on Pain Management	American Journal of Chinese Medicine	2020	71
7	Complementary and Alternative Medicine for Cancer Pain: An Overview of Systematic Reviews	Evidence-based Complementary And Alternative Medicine	2014	67
8	Chinese Herbal Medicine for Cancer Pain	Integrative Cancer Therapies	2007	59
9	Lamiophlomis rotata, an Orally Available Tibetan Herbal Painkiller, Specifically Reduces Pain Hypersensitivity States through the Activation of Spinal Glucagon-like Peptide-1 Receptors	Anesthesiology	2014	51
10	Medical use of cannabis products	Schmerz	2016	46

A total of 6,043 co-cited references were identified, and the top 13 are presented in Table 8. The co-cited references mainly consisted of systematic reviews, clinical guidelines, and studies evaluating complementary therapies for cancer pain, indicating that evidence-based pain management and integrative oncology constitute the intellectual foundation of this research field. The co-citation network is presented in Figure 7B. Overall, the citation and co-citation networks reveal the principal knowledge base and the most influential studies supporting the development of this research field.

**Table 8:** The top 13 most co-cited reference.

Rank	Title	Journal	Year	Citations
1	Prevalence of pain in patients with cancer: a systematic review of the past 40 years	Annals of Oncology	2007	8
2	Update on Prevalence of Pain in Patients With Cancer: Systematic Review and Meta-Analysis	Journal of Pain and Symptom Management	2016	7
3	Chinese Herbal Medicine for Cancer Pain	Integrative Cancer Therapies	2007	7
4	The effects of pilates on mental health outcomes: A meta-analysis of controlled trials	Complementary Therapies in Medicine	2015	6
5	A systematic review and meta-analysis on the use of traditional Chinese medicine compound kushen injection for bone cancer pain	Supportive Care in Cancer	2013	5
6	Efficacy of Complementary and Alternative Medicine Therapies in Relieving Cancer Pain: A Systematic Review	Journal of Clinical Oncology	2006	5
7	Trends in alternative medicine use in the United States, 1990–1997: results of a follow-up national survey.	JAMA	1998	5
8	Cerebrospinal fluid cytology in patients with cancer: minimizing false-negative results.	Cancer	1998	5
9	Complementary Alternative Treatments Used by Patients With Cancer in Eastern Turkey	Cancer Nursing	2003	5
10	East Asian Herbal Medicine to Reduce Primary Pain and Adverse Events in Cancer Patients: A Systematic Review and Meta-Analysis With Association Rule Mining to Identify Core Herb Combination	Frontiers in Pharmacology	2021	5
11	Multicenter, Double-Blind, Randomized, Placebo-Controlled, Parallel-Group Study of the Efficacy, Safety, and Tolerability of THC:CBD Extract and THC Extract in Patients with Intractable Cancer-Related Pain	Journal of Pain and Symptom Management	2009	5
12	Complementary/Alternative Medicine Use in a Comprehensive Cancer Center and the Implications for Oncology	Journal of Clinical Oncology	2000	5
13	Adult Cancer Pain, Version 3.2019, NCCN Clinical Practice Guidelines in Oncology	Journal Of The National Comprehensive Cancer Network	2019	5

### 3.6 Keywords analysis

Table 9 presents the most frequent keywords identified in this study. High-frequency keywords were primarily related to cancer pain, pain management, traditional Chinese medicine, acupuncture, evidence synthesis, and quality of life, indicating the major research themes in this field. These highlight a strong focus on pain management, particularly cancer and neuropathic pain. The prominence of terms like “traditional chinese medicine” and “acupuncture” underscores the integration of complementary therapies, while “systematic review” and “prevalence” reflect emphasis on evidence synthesis and epidemiological analysis.

**Table 9:** The top 24 keywords by frequency.

Rank	Keywords	TLS	Frequency	Rank	Keywords	TLS	Frequency
1	cancer pain	352	34	13	prevalence	72	9
2	acupuncture	171	17	14	quality-of-life	47	9
3	human	300	17	15	analgesia	108	8
4	pain	220	17	16	cancer	59	8
5	neuropathic pain	147	14	17	systematic review	107	8
6	herbal medicine	137	12	18	bone cancer pain	28	7
7	management	83	12	19	article	98	6
8	review	204	12	20	efficacy	38	6
9	chinese medicine	112	11	21	herbaceous agent	102	6
10	humans	181	10	22	morphine	69	6
11	traditional chinese medicine	85	10	23	opioids	34	6
12	chronic pain	66	9	24	pain management	75	6

**Figure 8:**
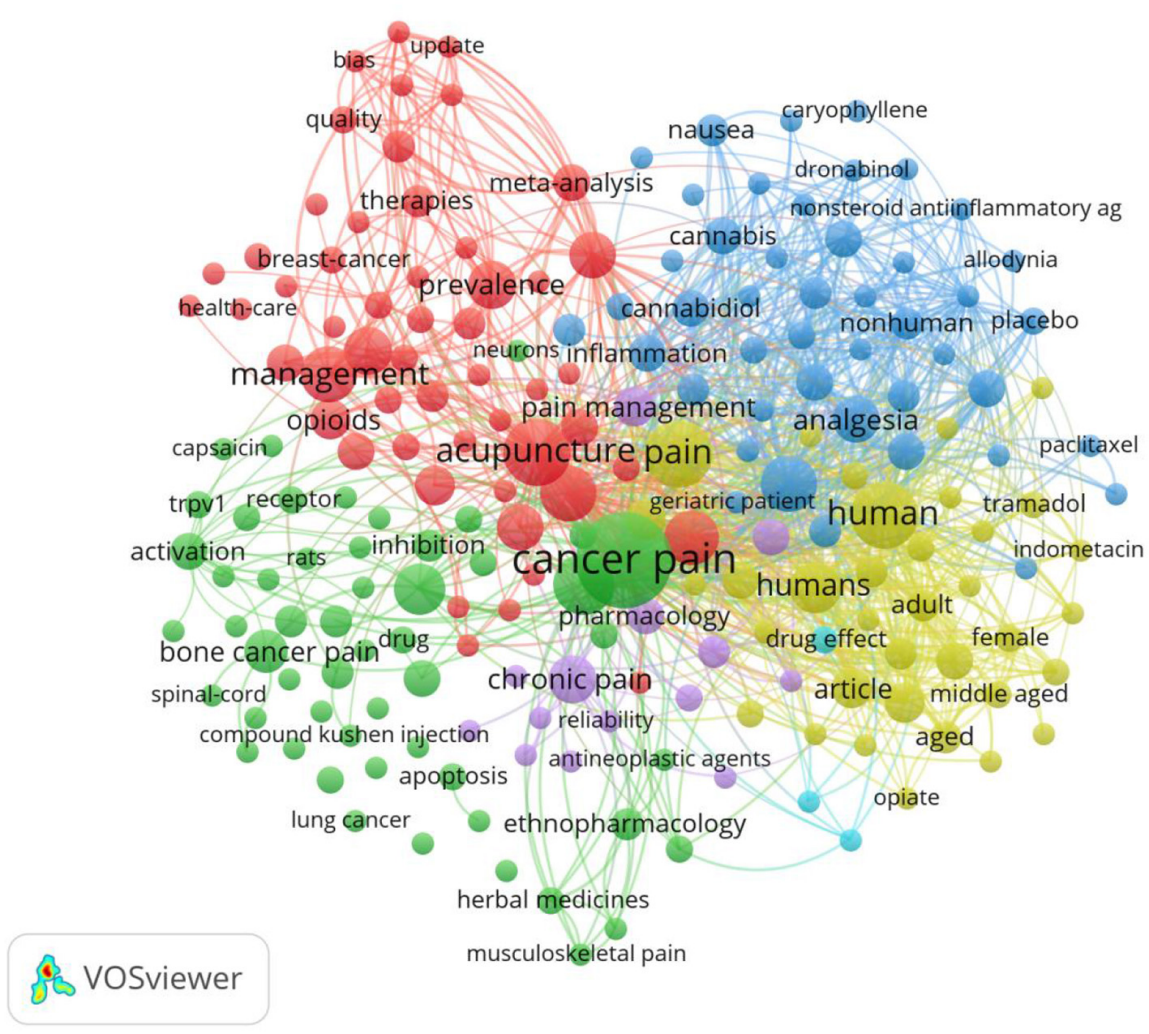
Network visualization of keywords. Each node represents a keyword, with node size proportional to its frequency of occurrence. Links indicate keyword co-occurrence relationships, and different colors represent thematic clusters identified by VOSviewer. The network illustrates the major research themes and the relationships among key topics, reflecting the evolution of research hotspots in Chinese herbal medicine for cancer pain management.

In this study, a co-occurrence network comprising 203 keywords (each appearing at least twice) was constructed and divided into five clusters using VOSviewer (Figure 8). The red cluster mainly comprised keywords related to cancer pain, acupuncture, pain management, opioids, and supportive care, highlighting research on the clinical management of cancer pain and the integration of Chinese herbal medicine with conventional analgesic strategies. The green cluster mainly comprised keywords related to traditional Chinese medicine, herbal medicine, apoptosis, inflammation, and bone cancer pain, highlighting mechanistic investigations and the pharmacological basis of Chinese herbal medicine for pain management. The blue cluster mainly comprised keywords related to analgesia, cannabinoids, nonsteroidal anti-inflammatory drugs, nausea, and placebo, highlighting pharmacological interventions and supportive therapies for cancer pain management. The yellow cluster mainly comprised keywords related to efficacy, systematic review, article, quality of life, and evidence synthesis, highlighting the growing emphasis on evidence-based evaluation and clinical assessment of Chinese herbal medicine interventions. The purple/cyan cluster mainly comprised keywords related to ethnopharmacology, herbal medicines, and complementary therapeutic approaches, highlighting research on the pharmacological basis and clinical application of herbal interventions for pain management. Collectively, these keyword patterns indicate that research is increasingly emphasizing evidence synthesis, integrative oncology, and mechanistic exploration, reflecting the evolving priorities of Chinese herbal medicine research for cancer pain management.

## 4 Discussion

Publication trends indicate that research on the use of Chinese herbal medicine for cancer pain management has gradually evolved from sporadic exploration to rapid expansion. Between 1998 and 2005, the number of publications was low and intermittent; from 2006 to 2013, it increased steadily; after 2014, it rose significantly, reaching a peak in 2025. The sustained increase in publications reflects growing research interest in this field. This trend may be linked to the growing clinical need for comprehensive cancer pain management and the potential complementary value of traditional Chinese medicine. However, an increase in publication volume does not necessarily imply sufficient high-quality evidence; future research should further strengthen methodological rigor, standardization of interventions, consistency of outcomes, and safety assessments. The decline observed in 2026 does not necessarily indicate a downward trend, as the data for that year is incomplete. First, the rise of integrative oncology has led to increasing attention in both academic and clinical circles toward treatment regimens that combine Chinese herbal medicine with Western medicine, providing new academic impetus for cancer pain research (26). Second, the growing demand among cancer patients and their families for pain relief and psychosocial support has driven an increase in the scientific validation and publication of Chinese herbal medicine adjunctive therapies (27). At the same time, the maturation of methodological approaches such as systematic reviews and clinical trials has enhanced the scientific rigor and reliability of research, attracting more scholars to the field (28). Furthermore, improvements in research policies and infrastructure have provided the necessary environmental and human resources to support Chinese herbal medicine research (5, 29). Taken together, these factors have collectively facilitated the rapid transition of Chinese herbal medicine from a niche area of exploration to mainstream research in the field of cancer pain management. More importantly, the observed publication trend has implications beyond increasing research output. It suggests that Chinese herbal medicine for cancer pain is gradually transitioning from an experience-based complementary therapy toward a more evidence-informed component of integrative oncology. The growing emphasis on randomized controlled trials, systematic reviews, and interdisciplinary collaboration reflects an increasing commitment to improving the quality and credibility of clinical evidence. This evolution is expected to facilitate the development of standardized treatment strategies, support future evidence-based clinical guidelines, and promote the broader integration of Chinese herbal medicine into multidisciplinary cancer pain management.

The analysis of contributing countries and institutions demonstrates that research on Chinese herbal medicine for cancer pain management is highly concentrated among a few leading nations, with the top 10 countries accounting for over 84% of all publications. China emerged as the most productive country, contributing 44 publications (41.90%), reflecting substantial investments in healthcare infrastructure, research funding, and growing scholarly interest in integrative medicine approaches (30). The United States, although publishing fewer papers (29, 27.62%), exhibits a high TLS, indicating its central role in fostering international collaboration and influencing global research directions (31). Visualization of the collaborative networks reveals that China and the United States occupy central positions within the international cooperation landscape. China's research contributions are closely linked with regional partners such as South Korea and Taiwan, as well as broader connections to Europe and Australia, illustrating a strong regional and intercontinental collaboration pattern. The United States, with an earlier start in this field, maintains extensive collaborative ties with multiple countries, highlighting its experience and academic influence. The dominance of China reflects its unique role as both the origin of Chinese herbal medicine and the primary source of clinical evidence in this field (32). In contrast, the high TLS of the United States reflects its established expertise in evidence-based oncology research and its active role in international scientific collaboration (31). From a clinical translation perspective, the close collaboration between Chinese institutions (with extensive clinical experience and patient cohorts) and US institutions (with rigorous trial methodologies) is particularly promising. This synergy can accelerate the design of culturally adaptable yet scientifically rigorous clinical protocols—for instance, integrating syndrome differentiation (traditional Chinese medicine diagnosis) with standardized Western outcome measures—thereby enhancing the clinical applicability of research findings across diverse healthcare settings.

At the institutional level, collaborations are similarly concentrated, with leading institutions from China (e.g., Shanghai University of Traditional Chinese Medicine, Beijing University of Chinese Medicine, and Fudan University) and the United States (e.g., University of Maryland and Memorial Sloan Kettering Cancer Center) serving as major hubs within the research network. This concentration is closely associated with differences in research capacity, clinical resources, and institutional expertise. Chinese institutions benefit from strong governmental support for the modernization and internationalization of Chinese herbal medicine, extensive clinical databases, and integrated hospital systems that facilitate large-scale clinical and translational studies (30). Leading Chinese universities, represented by Shanghai University of Traditional Chinese Medicine, have played important roles in promoting multicenter collaboration, clinical research, and the internationalization of Chinese herbal medicine research.

In contrast, major American institutions such as Memorial Sloan Kettering Cancer Center (MSKCC) have established internationally recognized integrative medicine programs that combine conventional oncology with evidence-based complementary therapies, including acupuncture and Chinese herbal medicine (33). The experience of MSKCC illustrates how comprehensive cancer centers can facilitate the integration of Chinese herbal medicine into evidence-based supportive oncology through rigorous clinical trial design, multidisciplinary collaboration, and international dissemination of research findings (34, 35). These institutions possess advanced clinical trial infrastructures, multidisciplinary oncology teams, and strong methodological expertise, which enable them to lead multicenter collaborations and high-quality evidence-based research. The strength of inter-institutional cooperation further underscores the importance of resource and expertise sharing, which accelerates research innovation, facilitates multicenter studies, and enhances the global impact of findings in this field (34, 35). International collaboration enables researchers to standardize protocols, recruit larger and more diverse patient populations, and improve the methodological rigor and reproducibility of clinical studies. Overall, these patterns indicate that international and inter-institutional collaboration is a critical driver of research development, enabling knowledge exchange and promoting the advancement of evidence-based applications of Chinese herbal medicine for cancer pain management.

The author analysis reveals a diverse and dynamic research community in the field of Chinese herbal medicine for cancer pain management. Key contributors, such as Lao, Li Xing (36) and Xu, Ling (37) not only lead in publication volume but also demonstrate high academic influence, as reflected by their TLS and citation counts. This highlights the distinction between sheer productivity and the broader impact of research, emphasizing that academic influence is more accurately captured by citation metrics and collaborative connectivity rather than publication numbers alone. The author collaboration network uncovers distinct thematic and regional research directions. Scholars affiliated with Chinese institutions primarily focus on clinical applications and integrative approaches to improve patient outcomes, optimize nursing practices, and enhance the quality of care through Chinese herbal medicine interventions (38). In contrast, Western scholars concentrate on methodological innovation, evidence synthesis, and the development of integrative treatment protocols. Notably, certain authors, such as Lao, Li Xing and Xu, Ling, act as central bridges in the network, fostering cross-institutional and international collaborations that facilitate knowledge exchange and dissemination of best practices. Co-citation analysis further identifies influential figures who, despite lower publication counts, occupy pivotal positions in the academic network due to high citation counts and strong collaborative ties. These hubs indicate that impactful scholarship often emerges from the depth and breadth of academic engagement, rather than volume alone. Overall, the author and co-citation networks highlight the interdisciplinary and cross-regional nature of research in this field, revealing opportunities to strengthen global collaboration, bridge geographical disparities, and promote integrated research strategies that enhance the effectiveness of Chinese herbal medicine in cancer pain management. More importantly, the emergence of influential authors and collaborative research groups reflects the gradual establishment of academic leadership in this field. By promoting multicenter studies, methodological innovation, and interdisciplinary collaboration, these researchers have strengthened the evidence base for Chinese herbal medicine and accelerated its translation into evidence-based cancer pain management.

The analysis of journals and co-cited journals demonstrates that research on Chinese herbal medicine for cancer pain management is concentrated in high-impact and interdisciplinary journals. Core journals such as Journal of Pain and Symptom Management (39), Frontiers in Pharmacology (40), and Integrative Cancer Therapies (41) are ranked in JCR Q1 or Q2 with high impact factors, indicating their significant academic influence and centrality in disseminating research findings. The prominence of these journals highlights their role in consolidating knowledge, providing platforms for evidence-based clinical studies, and shaping the research agenda within this field. Co-citation analysis further underscores the influence of journals that bridge disciplines, reflecting the integration of traditional medicine, clinical practice, pharmacology, and complementary therapies. Journals with high co-citation counts, such as Pain (42), Journal of Clinical Oncology (43), and Journal of Ethnopharmacology (44), serve as key nodes in the knowledge network, providing foundational references for both clinical and mechanistic studies. This pattern demonstrates that academic impact depends not only on publication volume but also on the journal's reach and its ability to connect research communities. Moreover, the findings highlight the importance of interdisciplinary collaboration, as journals at the intersection of medicine, pharmacology, and health informatics facilitate the exchange of methodologies, clinical protocols, and technological innovations. Such cross-disciplinary engagement promotes the application of Chinese herbal medicine in integrative cancer pain management, encourages methodological diversity, and enhances the global relevance and translation of research outcomes. Collectively, the journal and co-citation networks reflect a mature and interconnected academic ecosystem that supports both the consolidation of evidence and the continuous advancement of this field. These findings also provide useful guidance for researchers in selecting appropriate journals for literature retrieval and future manuscript submission.

The reference citation analysis highlights the foundational and highly influential studies that have shaped research on Chinese herbal medicine for cancer pain management. The most frequently cited works focus on clinical efficacy, complementary therapy integration, and methodological rigor, underscoring the field's emphasis on evidence-based practice. For instance, studies systematically reviewing Chinese herbal medicine interventions, evaluating patient outcomes, and exploring multi-component mechanisms serve as critical references for subsequent research (25). High co-citation frequency indicates that these references not only consolidate existing knowledge but also guide future research directions. Core studies address gaps in clinical application, standardization of treatment protocols, and integration with conventional therapies, providing both theoretical frameworks and practical guidance. The prominence of methodological literature, including systematic reviews and meta-analyses (45), reflects the field's commitment to rigorous study design and reliable evidence synthesis. Overall, the reference network demonstrates that research in this area is transitioning from exploratory investigations to more structured and practice-oriented scholarship. The evolution of cited references illustrates a trajectory characterized by clinical relevance, methodological standardization, and interdisciplinary integration, laying a robust foundation for advancing Chinese herbal medicine based interventions for cancer pain management globally. These highly cited references constitute the intellectual foundation of this field and may serve as key resources for researchers seeking to understand its development and future directions.

The co-occurrence analysis of keywords systematically reveals the thematic structure and evolving research trends in Chinese herbal medicine for cancer pain management. First, high-frequency keywords such as “cancer pain,” “pain,” “traditional Chinese medicine,” “herbal medicine” and “integrative oncology” highlight the central research focus on integrative pain management and symptom relief in oncology care. These findings suggest that the primary objective of this field is to explore complementary therapeutic approaches capable of enhancing analgesic efficacy, reducing opioid-related adverse effects, and improving patients' quality of life (46, 47). The frequent appearance of terms such as “neuropathic pain,” “bone cancer pain” and “quality of life” further indicates increasing attention toward specific pain syndromes and patient-centered outcomes in supportive cancer care (6). Second, keywords including “systematic review,” “meta-analysis,” “randomized controlled trial” and “evidence-based medicine” reflect the growing emphasis on clinical evidence synthesis and methodological rigor. This trend demonstrates that research in this field has gradually shifted from exploratory clinical observations toward evidence-based evaluation of Chinese herbal medicine efficacy and safety (6). Recent systematic reviews and meta-analyses have increasingly examined the effectiveness of oral and topical Chinese herbal medicine interventions in reducing pain intensity, improving analgesic duration, enhancing quality of life, and decreasing opioid-related complications (48). Moreover, recent studies have emphasized the role of Chinese herbal medicine as an adjuvant therapy in oncology supportive care, particularly for alleviating chemotherapy-related adverse effects and improving immune function (49). The increasing use of evidence synthesis methods also suggests a growing demand for standardized treatment protocols and higher-quality clinical evidence to support the integration of Chinese herbal medicine into modern oncology practice. In addition, the presence of keywords such as “opioids,” “adjuvant therapy,” “palliative care,” “integrative medicine” and “quality of life” demonstrates that Chinese herbal medicine is increasingly investigated as a complementary intervention alongside conventional cancer pain management strategies (46, 47). This reflects a broader movement toward multidisciplinary and integrative cancer care, where Chinese herbal medicine is studied not only for direct analgesic effects but also for symptom burden reduction, supportive care optimization, and improvement of patient wellbeing (49). Integrative oncology programs established in academic cancer centers have further promoted interdisciplinary collaboration and facilitated the incorporation of herbal medicine into supportive oncology care systems (50). The keyword clustering results further reveal several interconnected thematic directions. One major cluster focuses on clinical symptom management and therapeutic efficacy, emphasizing cancer pain relief and quality-of-life improvement. Another cluster centers on evidence evaluation and research methodology, highlighting systematic reviews, randomized controlled trials, and meta-analyses. A third cluster involves mechanistic and pharmacological investigations, including inflammation regulation, immune modulation, neuropathic pain pathways, and herbal compound activity (6, 49). Together, these clusters demonstrate the interdisciplinary nature of this field, integrating oncology, pharmacology, palliative medicine, and complementary medicine research. Collectively, these evolving research hotspots demonstrate that the field is shifting from empirical clinical application toward evidence-based, mechanism-oriented, and multidisciplinary research. This transition is expected to enhance the scientific credibility of Chinese herbal medicine, facilitate its incorporation into clinical guidelines, and promote its broader application in integrative cancer pain management. Clinically, these hotspots suggest that Chinese herbal medicine is being increasingly explored not only for analgesic effects, but also for opioid-sparing benefits, quality-of-life improvement, and supportive care optimization. Translationally, the growing attention to mechanisms, inflammation, network pharmacology, and specific pain subtypes suggests a need to link herbal formulations with active compounds, molecular targets, safety profiles, and clinically relevant cancer pain phenotypes.

Overall, this bibliometric study helps fill an important gap in the existing literature by providing a comprehensive and data-driven map of a field that has previously been examined mainly through clinical efficacy studies, intervention-specific reviews, or palliative care outcomes. By integrating publication trends, collaboration networks, knowledge structures, and evolving research hotspots, this study shows that research on traditional Chinese herbal medicine for cancer pain management has evolved from exploratory clinical practice toward evidence-based and integrative oncology research (51). For researchers, the identified research hotspots, collaboration patterns, and evolving knowledge structure provide valuable guidance for identifying research priorities and developing interdisciplinary collaborations. For clinicians, the growing evidence base reflects increasing interest in integrating Chinese herbal medicine into cancer pain management, while also highlighting the need for evidence-based evaluation before broader clinical implementation. However, the field still faces several structural challenges, including geographical disparities in research productivity, uneven academic influence, and insufficient international collaboration (46). These disparities may be associated with differences in healthcare systems, funding support, and the degree of acceptance of traditional medicine in different countries. Because Chinese herbal medicine is deeply rooted in traditional Chinese medicine theory and clinical practice, China maintains a dominant position in publication output and clinical application, whereas many Western countries still require stronger evidence regarding efficacy, safety, and standardization before widespread clinical adoption (52).

Future research should translate these bibliometric findings into clinically actionable priorities. The concentration of publications and collaboration hubs in China and the United States suggests that existing networks can be used to organize multinational, multicenter trials with standardized herbal interventions and harmonized outcomes, including pain intensity, opioid consumption, quality of life, adverse events, and safety monitoring. The prominence of evidence synthesis and clinical guideline-related references indicates the need to generate reproducible evidence that can support guideline development. Meanwhile, emerging hotspots such as network pharmacology, inflammation, and bone cancer pain suggest that mechanistic studies should validate active compounds, analgesic pathways, herb–drug interactions, and specific cancer pain phenotypes. Together, these directions may help bridge bibliometric trends with clinical translation and promote the evidence-based integration of Chinese herbal medicine into cancer pain management.

These priorities should be accompanied by stronger safety evaluation, quality control, and regulatory oversight. In particular, future studies should systematically assess herb–drug interactions, long-term safety, and reproducibility across different clinical settings. Such efforts will be essential for translating Chinese herbal medicine from empirical supportive care into standardized, evidence-based, and clinically applicable cancer pain management.

### 4.1 Limitations

Several limitations inherent to bibliometric methodology should be acknowledged when interpreting the results of this study. First, bibliometric analyses rely on publication and citation metrics, which reflect academic influence rather than the intrinsic quality of individual studies. Citation counts may be affected by multiple factors, including positive or negative citation motivations and self-citation practices. Therefore, highly cited publications should not be regarded as inherently higher-quality evidence. Second, the bibliometric data were retrieved only from the Web of Science Core Collection and Scopus. Although these databases provide standardized and high-quality citation information, they may not capture all relevant studies indexed in PubMed, Embase, CNKI, or other regional databases, potentially introducing selection bias. Third, only English-language publications were included, which may lead to language bias and underrepresent important studies published in other languages, particularly Chinese. Fourth, as the field of Chinese herbal medicine for cancer pain continues to evolve, newly published studies after the search date were not included and therefore the findings represent the knowledge landscape only up to the time of data collection. Finally, differences in healthcare systems, clinical practice, and regulatory environments across countries may limit the generalizability of the findings.

## 5 Conclusion

This bibliometric study shows that Chinese herbal medicine for cancer pain management is an emerging but rapidly expanding field. The bibliometric evidence indicates increasing publication activity, growing interdisciplinary engagement, and a gradual shift from exploratory clinical practice toward evidence-based evaluation, mechanistic exploration, and integrative oncology. China and the United States currently play leading roles, while influential institutions, authors, journals, and co-cited references have contributed to shaping the knowledge base of the field.

At the same time, the field remains unevenly developed. Research activity is geographically concentrated, collaboration networks are still relatively fragmented, and the available evidence is limited by methodological heterogeneity, insufficient high-quality multicenter trials, and inadequate standardization of herbal interventions, clinical outcomes, and safety assessment.

Future research should therefore move beyond simply increasing publication output and focus on generating clinically usable evidence. Priority should be given to international multicenter trials, standardized herbal interventions and outcome measures, rigorous safety evaluation, and mechanistic studies that link herbal compounds with specific cancer pain phenotypes. The key message of this bibliometric analysis is that Chinese herbal medicine has moved from empirical supportive use toward evidence-based integrative oncology, but its clinical translation will depend on whether future research can produce standardized, reproducible, and guideline-ready evidence for cancer pain management.

## Data Availability

The original contributions presented in the study are included in the article/Supplementary material, further inquiries can be directed to the corresponding authors.
