# Supplementary material for: Research hotspots and trends in the application of Chinese herbal medicine for cancer pain management: a bibliometric study
Source: Front Med (Lausanne). 2026 Jul 21;13:1889170. doi: 10.3389/fmed.2026.1889170 (PMC13434455; doi:10.3389/fmed.2026.1889170)
Supplement: Supplementary file 1 [file Table_1.DOCX]

Supplementary Material

Supplementary Table 1 Bibliographic information of the 105 publications included in the bibliometric analysis

| **NO** | **Title** | **Journal** | **First author** | **Year** |
| --- | --- | --- | --- | --- |
| 1 | Managing morphine-induced constipation: A controlled comparison of an Ayurvedic formulation and senna | Journal of Pain and Symptom Management | Ramesh, P. R. | 1998 |
| 2 | Prevalence and treatment of menopausal symptoms among breast cancer survivors | Journal of Pain and Symptom Management | Harris, P. F. | 2002 |
| 3 | A primer of complementary and alternative medicine and its relevance in the treatment of mental health problems | Psychiatric Quarterly | Mamtani, R. | 2002 |
| 4 | Case study no. 2: Tumor-related pain due to multiple myeloma | Deutsche Zeitschrift fur Akupunktur | Schmincke, Ch | 2004 |
| 5 | Efficacy of complementary and alternative medicine therapies in relieving cancer pain: A systematic review | Journal of Clinical Oncology | Bardia, A. | 2006 |
| 6 | Cancer Pain | Cancer Pain | de Leon-Casasola, O. A. | 2006 |
| 7 | Palliative care | Journal of Complementary Medicine | Anonymous | 2007 |
| 8 | Dietary supplement usage by patients with cancer undergoing chemotherapy: Does prognosis or cancer symptoms predict usage? | Journal of Supportive Oncology | Bardia, A. | 2007 |
| 9 | Influence of Chinese herb aitongke on the proliferation, apoptosis and Rb gene expression of hepatoma carcinoma cells HepG2 | Journal of Clinical Rehabilitative Tissue Engineering Research | Xu, B. | 2007 |
| 10 | Chinese herbal medicine for cancer pain | Integrative Cancer Therapies | Xu, L. | 2007 |
| 11 | Quality of reporting in 41 randomized controlled clinical trials of Chinese medicine for cancer pain | World Chinese Journal of Digestology | Xu, L. | 2007 |
| 12 | Cannabinoids in the management of difficult to treat pain | Therapeutics and Clinical Risk Management | Russo, E. B. | 2008 |
| 13 | Mechanisms of treatment of cancer pain with a topical Chinese herbal formula in rats | Chinese Medical Journal | Yu, S. | 2009 |
| 14 | Cancer pain control with traditional Chinese medicine | Supportive Cancer Care with Chinese Medicine | Bao, T. | 2010 |
| 15 | Compound Chinese herb for cancer pain: A systematic review | Chinese Journal of Evidence-Based Medicine | Du, Y. Q. | 2010 |
| 16 | Efficacy of acupuncture in treatment of cancer pain: A systematic review | Journal of Chinese Integrative Medicine | Peng, H. | 2010 |
| 17 | Progress in treatment for advanced gastric cancer | Chinese Journal of Clinical Oncology | Wang, J. | 2010 |
| 18 | Economic impact of potential drug-drug interactions in opioid analgesics | Journal of Medical Economics | Summers, K. H. | 2011 |
| 19 | Pharmacology of Opioids in the Treatment of Chronic Pain Syndromes | Pain Physician | Vallejo, R. | 2011 |
| 20 | Traditional Chinese medicine for cancer pain | Journal of Chinese Integrative Medicine | Wang, J. Y. | 2011 |
| 21 | High incidence and recurrence of transitional cell carcinoma in Taiwanese patients with end-stage renal disease | Nephrology | Wang, T. Y. | 2011 |
| 22 | Resveratrol, a neuroprotective supplement for Alzheimer's disease | Current Pharmaceutical Design | Li, F. | 2012 |
| 23 | Recent Non-Interventional Advances in Cancer Pain and Singaporean Patients | Annals Academy of Medicine Singapore | Saw, C. L. L. | 2012 |
| 24 | Topical treatment with Tong-Luo-San-Jie gel alleviates bone cancer pain in rats | Journal of Ethnopharmacology | Wang, J. Y. | 2012 |
| 25 | Advising Patients on the Use of Non-Herbal Nutritional Supplements During Cancer Therapy: A Need for Doctor-Patient Communication | Journal of Pain and Symptom Management | Ben-Arye, E. | 2013 |
| 26 | Cancer-Associated Pain and its Management | Pain Management in Veterinary Practice | Love, L. | 2013 |
| 27 | Complementary and Alternative Medicine for Cancer Pain: An Overview of Systematic Reviews | Evidence-Based Complementary and Alternative Medicine | Bao, Y. J. | 2014 |
| 28 | Use and Perceived Benefits of Complementary Therapies by Cancer Patients Receiving Conventional Treatment in Italy | Journal of Pain and Symptom Management | Bonacchi, A. | 2014 |
| 29 | Studies on the analgesic activities of Jia-Yuan-Qing pill and its safety evaluation in mice | Protoplasma | Tian, Y. | 2014 |
| 30 | Lamiophlomis rotata, an Orally Available Tibetan Herbal Painkiller, Specifically Reduces Pain Hypersensitivity States through the Activation of Spinal Glucagon-like Peptide-1 Receptors | Anesthesiology | Zhu, B. | 2014 |
| 31 | Topical treatment with Xiaozheng Zhitong Paste alleviates bone cancer pain by inhibiting proteinase-activated receptor 2 signaling pathway | Oncology Reports | Bao, Y. J. | 2015 |
| 32 | Traditional herbal medicine for cancer pain: A systematic review and meta-analysis | Complementary Therapies in Medicine | Lee, J. W. | 2015 |
| 33 | Transient Receptor Potential Channel and Interleukin-17A Involvement in LTTL Gel Inhibition of Bone Cancer Pain in a Rat Model | Integrative Cancer Therapies | Wang, J. Y. | 2015 |
| 34 | Cannabis for the Management of Pain: Assessment of Safety Study (COMPASS) | Journal of Pain | Ware, M. A. | 2015 |
| 35 | Medical use of cannabis products Lessons to be learned from Israel and Canada | Schmerz | Ablin, J. | 2016 |
| 36 | Combination therapy of gefitinib and korean herbal medicines could be a beneficial option for patients with non-small-cell lung cancer | Journal of Pharmacopuncture | Lee, K. | 2016 |
| 37 | The Cannabis sativa Versus Cannabis indica Debate: An Interview with Ethan Russo, MD | Cannabis and Cannabinoid Research | Piomelli, D. | 2016 |
| 38 | Pain and herbal medicine: Effectiveness of japanese kampo medicines on pains associated with cancer patients | Methods in Pharmacology and Toxicology | Uezono, Y. | 2016 |
| 39 | A Multicenter, Randomized, Double-Blind, Placebo-Controlled Trial of Shuangbai San for Treating Primary Liver Cancer Patients With Cancer Pain | Journal of Pain and Symptom Management | Ye, X. W. | 2016 |
| 40 | Application in anti-head and neck carcinoma of matrine compound injections | Fudan University Journal of Medical Sciences | Zheng, J. | 2016 |
| 41 | Chronic and refractory pain: A systematic review of pharmacologic management in oncology | Clinical Journal of Oncology Nursing | Brant, J. M. | 2017 |
| 42 | Chinese herb cinobufagin-reduced cancer pain is associated with increased peripheral opioids by invaded CD3/4/8 lymphocytes | Oncotarget | Chen, T. | 2017 |
| 43 | Complementary and Alternative Medicine in Oncology-WhatÂ Makes Sense? | Deutsche Medizinische Wochenschrift | Eustachi, A. | 2017 |
| 44 | Value and Feasibility of Auricular Point Sticking with the Medicinal Herb Seed in Relieving Cancer Pain | Chinese General Practice | Gu, L. L. | 2017 |
| 45 | Capsicum: A Natural Pain Modulator | Nutritional Modulators of Pain in the Aging Population | Kulkarni, Y. A. | 2017 |
| 46 | Inhibitory Effect of Viola odorata Extract on Tumor Growth and Metastasis in 4T1 Breast Cancer Model | Iranian Journal of Pharmaceutical Research | Alipanah, H. | 2018 |
| 47 | Cannabinoids and cancer pain: A new hope or a false dawn? | European Journal of Internal Medicine | Brown, M. R. D. | 2018 |
| 48 | Thematic trends in complementary and alternative medicine applied in cancer-related symptoms | Journal of Data and Information Science | Moral-Munoz, J. A. | 2018 |
| 49 | Chinese herbal medicine for opioid induced constipation in cancer patients: Protocol for a systematic review | Medicine (United States) | Qi, S. | 2018 |
| 50 | Ultra-Liquid Chromatography Tandem Mass Spectrometry (UPLC-MS/MS)-Based Pharmacokinetics and Tissue Distribution Study of Koumine and the Detoxification Mechanism of Glycyrrhiza uralensis Fisch on Gelsemium elegans Benth | Molecules | Wang, L. | 2018 |
| 51 | 8-O-Acetyl Shanzhiside Methylester From Lamiophlomis Rotata Reduces Neuropathic Pain by Inhibiting the ERK/TNF-α Pathway in Spinal Astrocytes | Frontiers in Cellular Neuroscience | Zhang, W. | 2018 |
| 52 | Cannabinoids versus placebo or no intervention for pain: protocol for a systematic review with meta-analysis and trial sequential analysis | Bmj Open | Barakji, J. A. | 2019 |
| 53 | Yokukansan Alleviates Cancer Pain by Suppressing Matrix Metalloproteinase-9 in a Mouse Bone Metastasis Model | Evidence-Based Complementary and Alternative Medicine | Nakao, K. | 2019 |
| 54 | Epidiolex and potential derived drugs from Cannabis and their future prospectives | Medicinal Plants | Rajpal, C. | 2019 |
| 55 | Complementary and Alternative Medicine for Breast Cancer Patients: An Overview of Systematic Reviews | Yakugaku Zasshi-Journal of the Pharmaceutical Society of Japan | Sasaki, Y. | 2019 |
| 56 | Roles of Cannabinoids in Melanoma: Evidence from In Vivo Studies | International Journal of Molecular Sciences | Bachari, A. | 2020 |
| 57 | A Systematic Review of CheeZheng Pain Relieving Plaster for Musculoskeletal Pain: Implications for Oncology Research and Practice | Integrative Cancer Therapies | Chen, C. | 2020 |
| 58 | A Scoping Review on Clinical Trials of Pain Reduction With Cannabis Administration in Adults | Journal of Clinical Medicine Research | Haleem, R. | 2020 |
| 59 | Network Pharmacology-Based Study on the Molecular Biological Mechanism of Action for Compound Kushen Injection in Anti-Cancer Effect | Medical Science Monitor | He, R. R. | 2020 |
| 60 | An Investigation of the Molecular Mechanisms Underlying the Analgesic Effect of Jakyak-Gamcho Decoction: A Network Pharmacology Study | Evidence-Based Complementary and Alternative Medicine | Lee, H. S. | 2020 |
| 61 | Effect of Siegesbeckiae Herba in treating chronic pain | Zhongguo Zhongyao Zazhi | Lin, Y. Y. | 2020 |
| 62 | Effects of Herbal Medicines on Pain Management | American Journal of Chinese Medicine | Luo, Y. | 2020 |
| 63 | Identification of herbal categories active in pain disorder subtypes by machine learning help reveal novel molecular mechanisms of algesia | Pharmacological Research | Xu, X. | 2020 |
| 64 | α-Cyperone inhibitory effects on tumor-derived DNA trigger microglia by STING pathway | Journal of Ethnopharmacology | Gao, P. | 2021 |
| 65 | Implementation of medicinal cannabis in Australia: innovation or upheaval? Perspectives from physicians as key informants, a qualitative analysis | Bmj Open | Hallinan, C. M. | 2021 |
| 66 | East Asian herbal medicine for cancer pain: A protocol for systematic review and meta-analysis with using association rule analysis to identify core herb pattern | Medicine (United States) | Jo, H. G. | 2021 |
| 67 | Traditional Chinese medicine in integrative oncology: Study situation and fields of application | Gynakologe | Klose, P. | 2021 |
| 68 | Molecular evidence of compound kushen injection against lung cancer: A network pharmacology-based investigation from western medicine to traditional medicine | Anti-Cancer Agents in Medicinal Chemistry | Liang, S. X. | 2021 |
| 69 | Herbal Topical Analgesic for Pain Management: Perspectives from Cancer Patients | Pain Medicine | Liou, K. T. | 2021 |
| 70 | Ojeok-san ameliorates visceral and somatic nociception in a mouse model of colitis induced colorectal cancer | Plos One | Cunningham, P. | 2022 |
| 71 | Improved Therapeutic Efficacy of CBD with Good Tolerance in the Treatment of Breast Cancer through Nanoencapsulation and in Combination with 20(S)-Protopanaxadiol (PPD) | Pharmaceutics | Fu, J. X. | 2022 |
| 72 | Medical nutrition therapy for chronic pain management | Clinical Pain Management: A Practical Guide, Second Edition | Glenn, A. | 2022 |
| 73 | Editorial: Herbal Medicines in Pain Management | Frontiers in Pharmacology | Hu, W. L. | 2022 |
| 74 | East Asian Herbal Medicine to Reduce Primary Pain and Adverse Events in Cancer Patients : A Systematic Review and Meta-Analysis With Association Rule Mining to Identify Core Herb Combination | Frontiers in Pharmacology | Jo, H. G. | 2022 |
| 75 | Non-pharmacological integrative therapies for chronic cancer pain | Journal of Oncology Pharmacy Practice | Katta, M. R. | 2022 |
| 76 | Chinese Medicinal Herb-Derived Carbon Dots for Common Diseases: Efficacies and Potential Mechanisms | Frontiers in Pharmacology | Li, D. | 2022 |
| 77 | Research Progress in the Pharmacological Activities, Toxicities, and Pharmacokinetics of Sophoridine and Its Derivatives | Drug Design, Development and Therapy | Tang, Q. | 2022 |
| 78 | Medical Cannabis Is Not Associated with a Decrease in Activities of Daily Living in Older Adults | Biomedicines | Abuhasira, R. | 2023 |
| 79 | Ginger (Zingiber officinale) and Biomedical Applications to Cancer: Cellular and Molecular Targets of Extracts | Ancient and Traditional Foods, Plants, Herbs and Spices used in Cancer | Ghareghomi, S. | 2023 |
| 80 | Complementary and Alternative Strategies for Cancer Prevention and Therapy | Functional Foods for Health Maintenance: Understanding their Role in Cancer Prevention | Gupta, S. | 2023 |
| 81 | Cannabis-based medicines and medical cannabis for adults with cancer pain | Cochrane Database of Systematic Reviews | Häuser, W. | 2023 |
| 82 | Buprenorphine-Naloxone in the Setting of Kratom Withdrawal, Opioid Use Disorder, and Stage IV Lung Adenocarcinoma | Journal of Palliative Medicine | Hong, S. | 2023 |
| 83 | A comprehensive and mechanistic review on protective effects of kaempferol against natural and chemical toxins: Role of NF-κB inhibition and Nrf2 activation | Biofactors | Hosseini, A. | 2023 |
| 84 | Biological Functions of TRPV1 | Progress in Biochemistry and Biophysics | Liu, X. Y. | 2023 |
| 85 | Topical Traditional Chinese Medicines for Cancer Pain: A Systematic Review and Meta-analysis of Randomized Controlled Trials | Integrative Cancer Therapies | Liu, Y. H. | 2023 |
| 86 | Cannabinoids in Treating Chemotherapy-Induced Nausea and Vomiting, Cancer-Associated Pain, and Tumor Growth | International Journal of Molecular Sciences | Bathula, P. P. | 2024 |
| 87 | Editorial: Herbal medicines in pain management, volume II | Frontiers in Pharmacology | Delfino, D. V. | 2024 |
| 88 | Diversities of Omnipotent Tulsi: Pharmacological and Chemical Aspects | The Chemistry inside Spices and Herbs: Research and Development: Volume 3 | Dobhal, K. | 2024 |
| 89 | Cancer Pain Management with Traditional Chinese Medicine: Current Status and Future Perspectives | American Journal of Chinese Medicine | Ji, J. F. | 2024 |
| 90 | Addiction stigma and opioid use in chronic non-cancer pain management in Nigeria | Sociology of Health & Illness | Nelson, E. U. E. | 2024 |
| 91 | Integrative Medicine for Cancer-Related Pain: A Narrative Review | Healthcare | Samuels, N. | 2024 |
| 92 | Liu-Shen-Wan inhibits PI3K/Akt and TRPV1 signaling alleviating bone cancer pain in rats | Cancer Biology & Therapy | Zhang, H. | 2024 |
| 93 | Advancements in Non-Addictive Analgesic Diterpenoid Alkaloid Lappaconitine: A Review | International Journal of Molecular Sciences | Zhang, W. | 2024 |
| 94 | Oxygen-Generating Biomimetic Nano-Herb System for Synergistic Therapy & Pain Relief in Triple-Negative Breast Cancer via HIF-1α/VEGF Pathway | International Journal of Nanomedicine | Cao, D. C. | 2025 |
| 95 | Combination of acupoint herbal plaster and ear acupuncture for effective pain relief in patients after laparoscopic appendectomy | Pakistan Journal of Pharmaceutical Sciences | Huang, H. | 2025 |
| 96 | A Randomized, Double-Blind, Placebo-Controlled Trial Protocol Using the Fuzhengxiaoliu Patch for the Management of Primary Liver Cancer Pain | Journal of Pain Research | Li, S. M. | 2025 |
| 97 | Theoretical and practical advantages of the “Brain-Heart-Kidney-Essence Chamber” axis in prostate cancer | Chinese Science Bulletin | Li, Y. | 2025 |
| 98 | Angelica gigas Nakai (Korean Dang-gui) Root Alcoholic Extracts in Health Promotion and Disease Therapy - active Phytochemicals and In Vivo Molecular Targets | Pharmaceutical Research | Lü, J. X. | 2025 |
| 99 | Evaluation of In Vitro and In Vivo Pharmacological Activity of Elatostema sessile With In Silico Approaches | Food Science & Nutrition | Nath, A. K. | 2025 |
| 100 | Luteolin alleviates lung cancer pain in mice by modulating AKT1 | Turkish Journal of Biochemistry-Turk Biyokimya Dergisi | Tu, H. Y. | 2025 |
| 101 | Unconventional Treatments for Pancreatic Cancer: A Systematic Review | Cancers | Vella, R. | 2025 |
| 102 | Chinese herbal medicine for chronic pain: a bibliometric analysis based on integrated databases (2011-2024) | Frontiers in Medicine | Wang, X. Y. | 2025 |
| 103 | Progress in Research on Alleviating the Symptoms Associated With Advanced Cancer Using Traditional Chinese Medicine | Pain Research & Management | Jiao, C. M. | 2026 |
| 104 | Oral Chinese herbal medicine in combination with opioids for treatment of cancer pain: A systematic review and meta-analysis | Journal of Traditional and Complementary Medicine | Le, T. C. | 2026 |
| 105 | Efficacy of Acupuncture Combined with the Three-Step Analgesic Protocol in Treating Pain in Liver Cancer Pain: A Bayesian Network Meta-Analysis | Journal of Pain Research | Li, T. | 2026 |
